# 5-Azacytidine promotes invadopodia formation and tumor metastasis through the upregulation of PI3K in ovarian cancer cells

**DOI:** 10.18632/oncotarget.18580

**Published:** 2017-06-20

**Authors:** Dan Cao, Dan Li, Yong Huang, Yu Ma, Binglan Zhang, Chengjian Zhao, Senyi Deng, Min Luo, Tao Yin, Yu-Quan Wei, Wei Wang

**Affiliations:** ^1^ Department of Abdominal Oncology, Cancer Center and State Key Laboratory of Biotherapy, Collaborative Innovation Center for Biotherapy, West China Hospital, Sichuan University, Chengdu, Sichuan, China; ^2^ Department of Gynecology, West China Second Hospital, Sichuan University, Chengdu, Sichuan, China

**Keywords:** 5-Azacytidine, methylation, invadopodia, metastasis, ovarian cancer

## Abstract

The high incidence of metastasis accounts for most of the lethality of ovarian cancer. Invadopodia are small, specialized types of machinery that degrade the extracellular matrix and are thus involved in the invasion and metastasis of cancer cells. The formation of invadopodia is regulated by both genetic and epigenetic factors. However, the ways by which methylation/demethylation regulates the dynamics of invadopodia in ovarian cancer are largely unknown. In this study, we found that the inhibition of methylation by 5-AZ (5-Azacytidine) increased the formation of invadopodia and enhanced degradation of the extracellular matrix in ovarian cancer cells. In mouse xenograft models, treatment with 5-AZ increased the number of metastatic nodules, which suggests an elevated potential for metastasis by demethylation. Further investigation indicated that the inhibition of methylation elevated the transcription of PIK3CA and upregulated genes involved in the PI3K-AKT signaling pathway. In addition, this induction likely occurs though the epigenetic regulation of PIK3CA because analyses of the DNA methylation level of the PIK3CA promoter region found that 5-AZ treatment decreased the methylation of CpG islands in SKOV3 and A2780 cells. Our study demonstrated that epigenetic factors regulate the metastatic potential of ovarian cancer cells and provide rationale for therapies that inhibit PI3K- invadopodia-mediated metastasis.

## INTRODUCTION

Ovarian cancer is the most lethal gynecologic malignancy in the world [1]. Epithelial ovarian cancer (EOC), which comprises approximately 90% of ovarian cancers, is believed to arise from the ovarian surface epithelium (OSE) or from the fallopian tube fimbriae [2]. Approximately 75% of cases of EOC are diagnosed at advanced stages (III-IV) and require a combination of cytoreductive surgery and chemotherapy. Although most patients respond to platinum-based chemotherapy, relapses and metastasis are common, which results in a 5-year survival rate below 25% [3]. Peritoneal metastasis is the main cause of medical therapy failure and cancer-related death in patients with EOC. The metastasis of EOC involves a distinctive metastatic process, which rarely involves the hematogenous route that is commonly observed in other cancers. The cancer cells exfoliate from the surface of the ovary and subsequently attach to peritoneal surfaces where they invade to form metastatic tumors. This mode of metastasis places unique demands on ovarian cancer cells and may require a specific molecular mechanism that differs from hematogenous metastasis [4]. Therefore, there is an urgent need for a better understanding of the mechanisms of metastasis to develop novel therapeutic strategies to treat EOC.

Accumulating evidence has demonstrated that cancer cells overcome the barrier to metastatic spread via the formation of specialized membrane structures called invadopodia and these structures mediate the degradation of the ECM, which results in invasion and metastasis [5]. Cellular migration and invasion though tissue barriers is important for a number of physiological and pathological conditions, including the dissemination of cancer cells during metastasis. The extracellular matrix (ECM), which is assembled from proteoglycans and fibrous proteins, is a key barrier to cell invasion. Actin-based subcellular structures termed invadopodia are now well-characterized as specialized machinery that function in ECM degradation [6]. Many human cancer cells can form invadopodia, including breast cancers, melanoma, squamous cell carcinomas of the head and neck, glioblastomas, and ovarian cancer [7]. One ECM protein in particular, fibronectin, is one of the most abundant proteins of the ECM and of the basement membrane in human omentum and peritoneum [8], which are the most common sites of ovarian cancer metastasis. Various studies have found correlations between the ability of cells to form invadopodia and invasiveness and metastasis *in vitro* and *in vivo* in EOC [9, 10]. Recent studies have demonstrated that invadopodia might be regulated by some pivotal factors that are present in EOC cells that ultimately result in invadopodia-mediated metastasis [11, 12].

It is widely accepted that the multistep process of cancer evolution is driven by both genetic and epigenetic abnormalities [13]. Unlike genetic alterations, epigenetic changes are potentially reversible, which makes them attractive and promising targets for therapeutic intervention. The epigenetic regulation of DNA-templated processes has been widely studied over the last 10 years. DNA promoter methylation, histone modification, nucleosome remodeling, and RNA-mediated targeting regulate many biological processes that are fundamental to the genesis of cancer [14]. Epigenetic gene dysregulation is associated with tumor formation and progression to a malignant stage. Previous studies have indicated that abnormal DNA methylation, a major epigenetic modification, is involved in the dysregulation of the cell cycle and apoptosis as well as in the proliferation and differentiation of tumor cells. In addition, abnormal methylation of DNA promoter regions including the hypomethylation of oncogenes and the methylation of tumor suppressor genes(TSGs) related to metastasis enhance metastatic behavior in cancer cells; consequently, DNA hypomethylating agents can restore the normal methylation process [15–17]. However, a recent study has shown that hypomethylating agents enhance tumor cell invasion and metastasis through a transcription-dependent modulation of matrix metalloprotease-1 (MMP-1) expression [18].

Therefore, this study focuses on whether the status of DNA methylation is related to invasion and metastasis and whether methylation regulates invadopodia formation in EOC.

## RESULTS

### 5-AZ suppresses tumor growth but promotes metastasis

**Figure 1:**
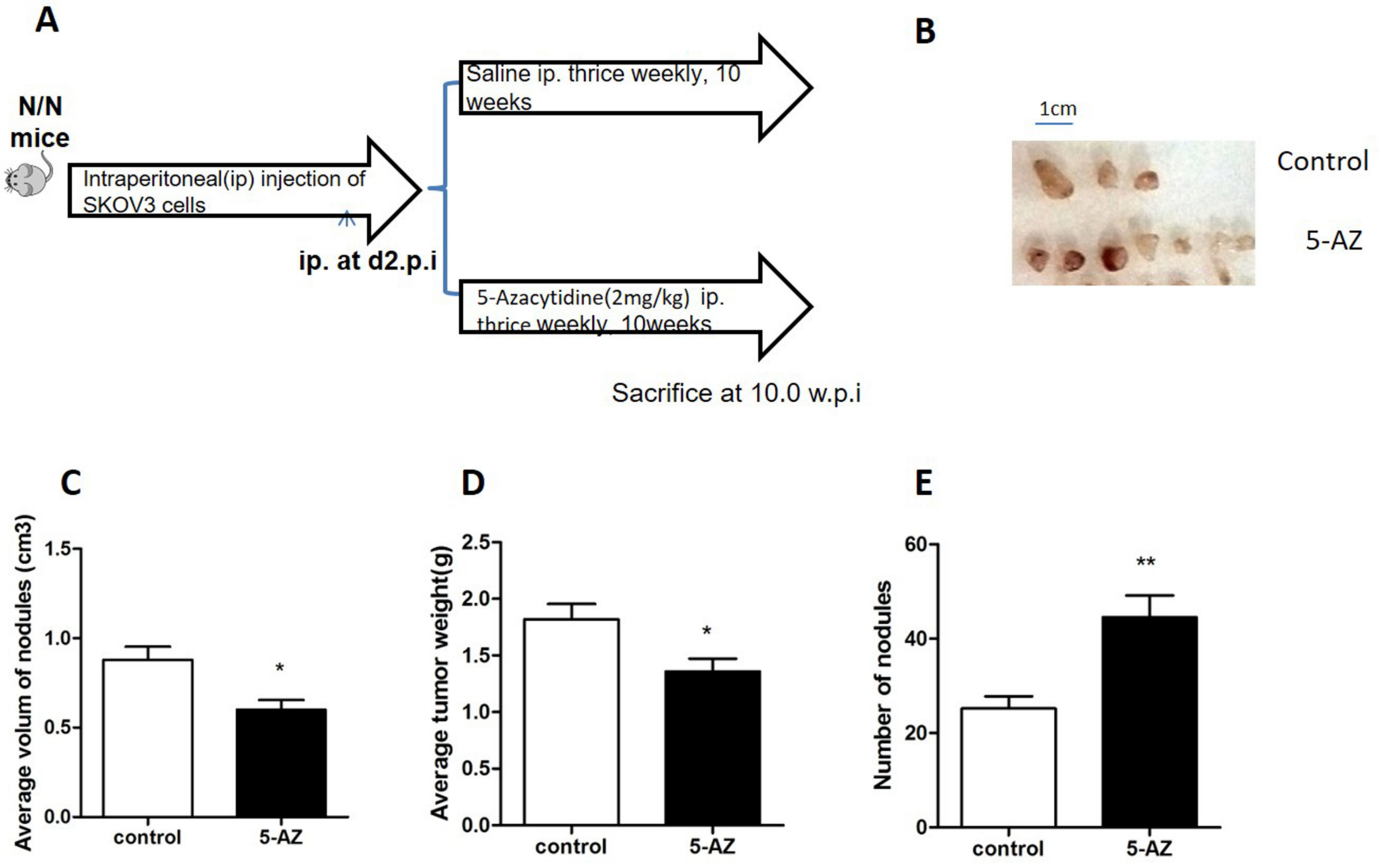
5-AZ treatment promotes invasion and metastasis in the mouse peritoneum (**A**) Study design. (**B**) Gross tumor nodule on the peritoneal. (**C**) The average volume of each nodule on the peritoneal surfaces was measured 10 weeks after the injection of saline(control) or 5-AZ in SKOV3 cell xenografts (*P* = 0.0348). (**D**) Average tumor weight was calculated between the two group (*P* = 0.0185). (**E**) The number of invasive and metastatic nodules was counted in the 5-AZ treatment group and in the control group (*P* = 0.0079). Student's *t*-test: mean ± S.E. *n* = 5, *P < 0.05 **P < 0.01.

Originally, we had planned to treat ovarian cancer with 5-AZ, a demethylating agent that is typically used to treat patients with melanoma and leukemia. To test the efficacy of 5-AZ on ovarian cancer, SKOV3 xenografts were generated by IP injection into nude mice. The mice were treated with 2 mg/kg 5-AZ or with normal saline thrice weekly for 10 weeks starting from the second day after injection (Figure 1A). In the 5-AZ treated and control groups, metastatic nodules were seen in peritoneum, liver, spleen and intestine (Figure 1B). In the 5-AZ treated mice, metastatic nodules even were seen in lung. Interestingly, the number of metastatic nodules was increased (Figure 1E) and the volume and weight per nodule were decreased (Figure 1C, 1D), when compared with the control group. These data suggest that 5-AZ inhibits growth of tumors but promotes the metastasis.

### *5*-AZ increases invadopodia formation in ovarian and breast cancer cells

**Figure 2:**
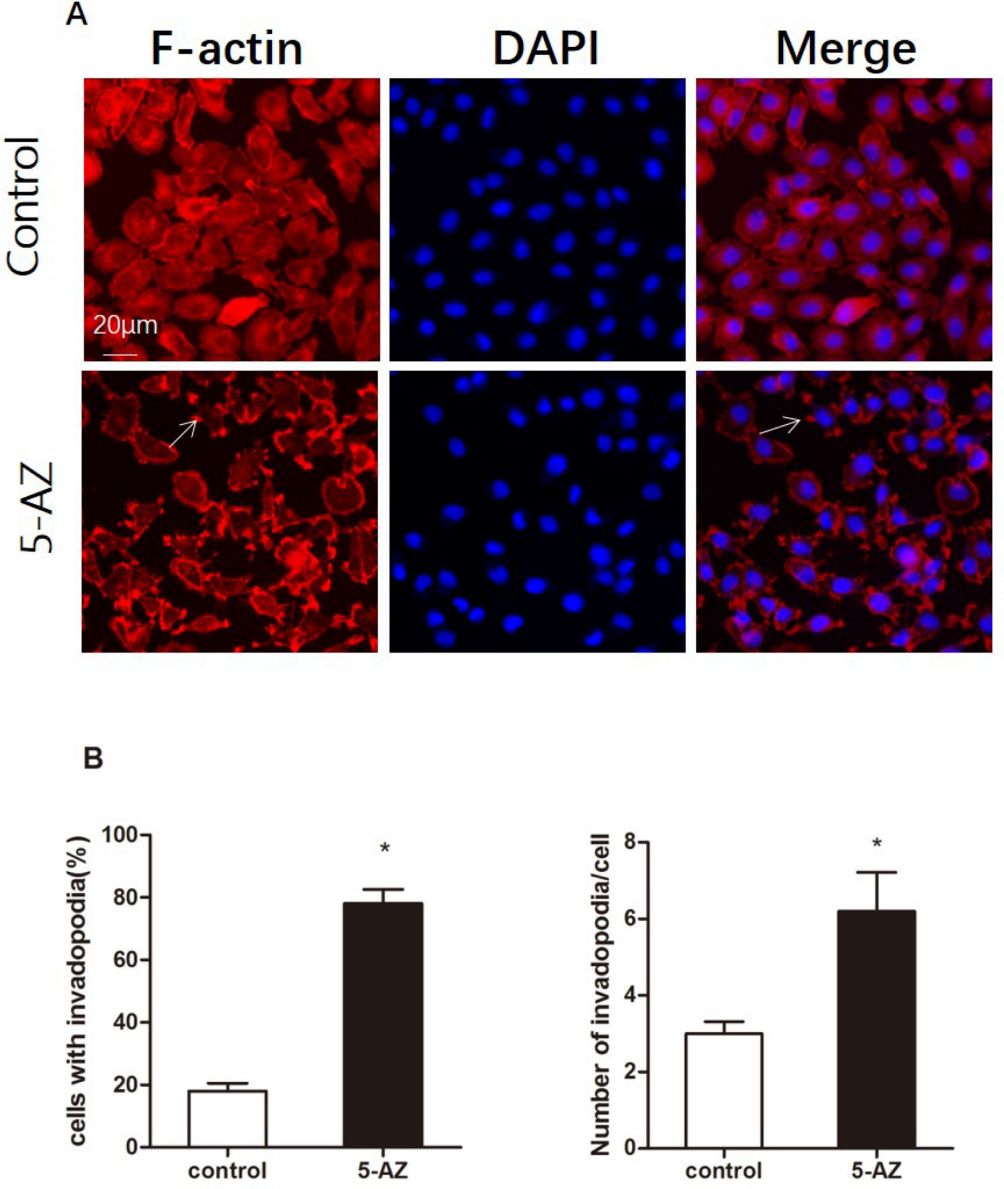
5-AZ promotes the formation of invadopodia in SKOV3 cells (**A**) Representative immunofluorescence images show localization of DAPI(blue) and F-actin (red) as well as an overlay of both channels (merge) in SKOV3 cells. Arrows show the positions of actin dots that are localized to a pericellular pool. SKOV3 cells were plated and grown in 24-well optical plates for 1 day, incubated with DMSO (upper panel) or 5-AZ (30 μM) (lower panel) for 48 h and then assayed for invadopodium formation by F-actin staining. The number of invadopodia largely increased in the 5-AZ treatment group. (**B**) Quantitation of the invadopodia that formed: invadopodia-positive cells (left panel) and the number of invadopodia per cell (right panel) in SKOV3 cells that were treated with 5-AZ. Student's *t*-test: mean ± S.E. *n* = 3. *P < 0.05. vs. control (untreated cells)

**Figure 3:**
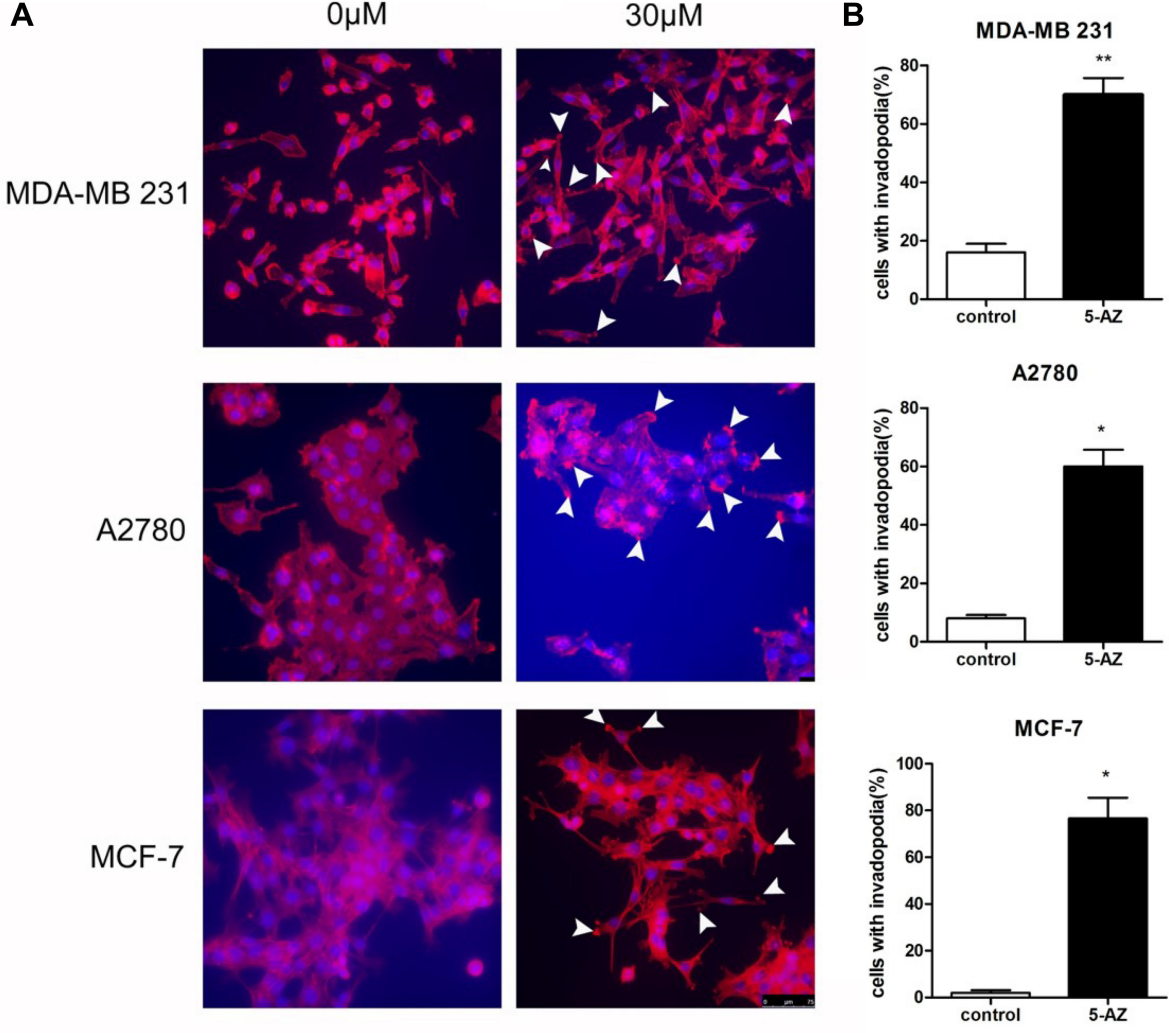
5-AZ increased the formation of invadopodia in cells of MDA-MB 231, A2780 and MCF-7 (**A**) Invadopodia largely increased after 5-AZ treatment as arrows show the F-actin location. (**B**) Quantitation of cells with invadopodia in the cell lines that were treated with 5-AZ shows that 5-AZ promoted invadopodia formation in both ovarian and breast cancer cells *in vitro*. Student's *t*-test: mean ± S.E. *n* = 3. *P < 0.05. **P < 0.01 vs. control (untreated cells)

Distant metastasis is a complex process that involves extravasation from the original site as well as intravasation and localization to metastatic sites. Degradation of the ECM that surrounds the tumor cells is a prerequisite for the extravasation process. Invadopodia-mediated ECM degradation probably promotes metastasis in ovarian cancer. Thus, we wondered whether the induction of metastasis by 5-AZ is mediated by invadopodia. To evaluate the effect of 5-AZ on ovarian cancer cells, SKOV3 cells were plated and grown in 24-well optical plates for 1 day and incubated with for 5-AZ (30 μmol/l) 48 h. This concentration is documented as IC50 of 5-AZ to SKOV3 cells (Supplementary Figure 1). The cells were then fixed and stained with 4′,6-diamidino-2-phenylindole (DAPI) and phalloidin to visualize the nuclei and F-actin, respectively (Figure 2A). SKOV3 cell growth was inhibited by 5-AZ (Supplementary Figure 2), but invadopodia formation was increased in the cells treated with 5-AZ. We subtracted the image background for the red channel (F-actin), and the nucleus of each cell was identified from the DAPI images. The number of detected fluorescent “aggregates” represented rosettes of invadopodia and was reported for each cell object as the “Number of Detected Rosettes per Cell” parameter. The average number of detected rosettes of invadopodia per cell and the number of cells with invadopodia were calculated for each well. 5-AZ increased both the fraction of cells that were positive for invadopodia and the number of invadopodia per cell (Figure 2B). In addition, other 3 cell lines, MDA-MB 231, A2780 and MCF7, were treated with 5-AZ. As shown in Figure 3, 5-AZ treatment, same concentration and treating time as that in SKOV3, increased the formation of invadopodia in these cells (Figure 3). These data indicated that 5-AZ increases the formation of invadopodia in ovarian and breast cancer cells.

### 5-AZ increases the ECM degradation function of invadopodia in ovarian cancer cells

**Figure 4:**
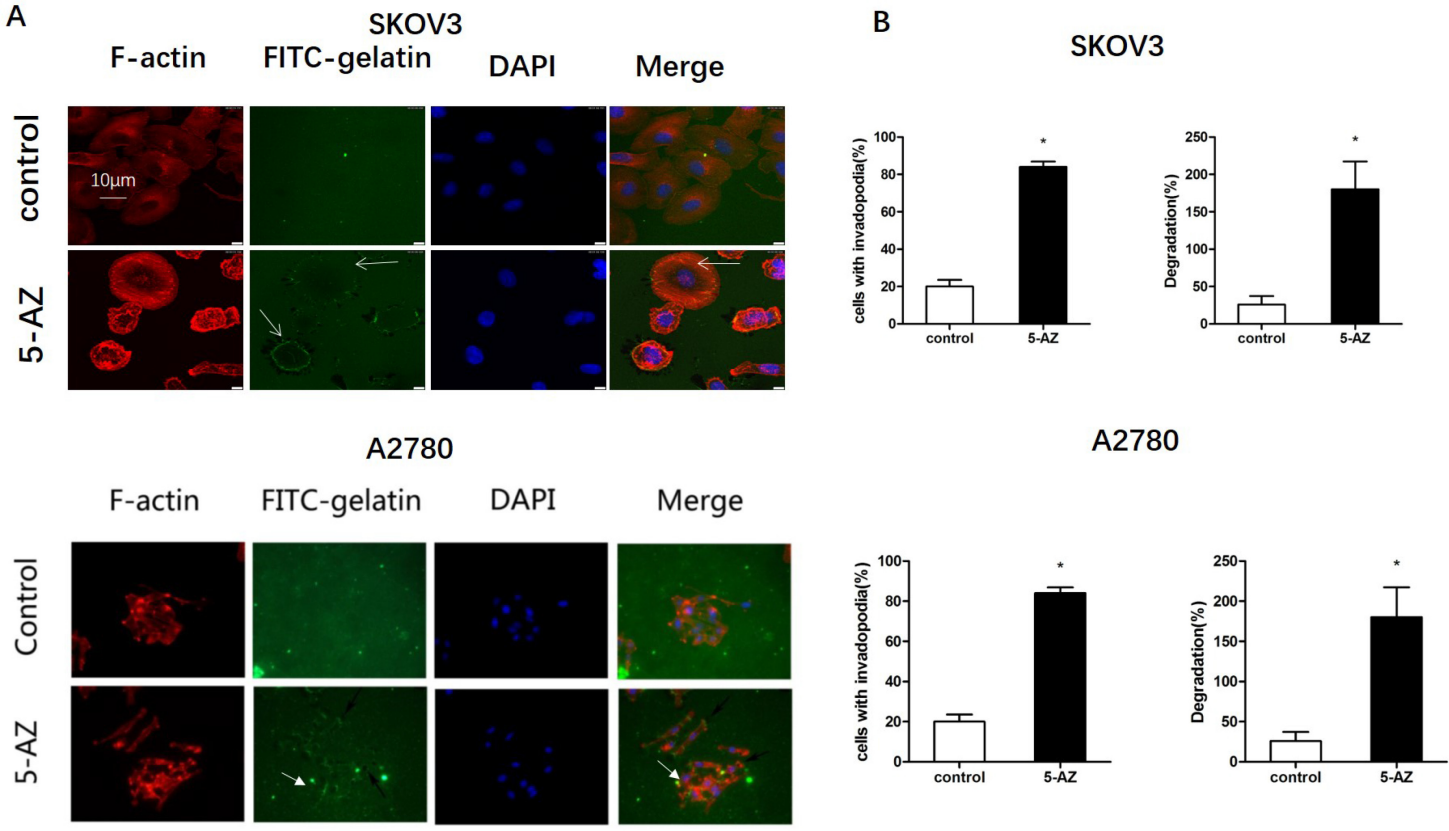
Effect of 5-AZ on the function of invadopodia in SKOV3 and A2780 cells (**A**) Representative immunofluorescence images show the localization of FITC-gelatin (green), F-actin (red) and DAPI (blue) in SKOV3 and A2780 cells. Arrows indicate the positions of invadopodia according to F-actin staining and gelatin degradation. FITC-labeled gelatin-coated coverslips were prepared and SKOV3 cells were cultured for 24 h. The coverslips with attached cells were subsequently processed using standard fluorescence microscopy procedures. The degradation of the gelatin was clearly enhanced in the 5-AZ treatment group. (**B**) Quantification of FITC-gelatin degradation (percent of degraded areas normalized to the cell number) in SKOV3 (upper panel) and A2780 (lower panel) cells. Student's *t*-test: mean ± S.E. *n* = 3. *P < 0.05. vs. control (untreated cells).

To determine if the function of invadopodia increased with 5-AZ treatment, we next tested the effect of 5-AZ on the ability of SKOV3 and A2780 cells to degrade a film of fluorescently labeled gelatin. The degradation of fluorescein isothiocyanate (FITC)-labeled gelatin was increased after treatment with 5-AZ for 24 h in two cell lines (Figure 4A). To test the effects of 5-AZ on the invasiveness of human ovarian cancer cells, we used SKOV3 and A2780 cells in which the quantification of the number of invadopodia is facilitated by the relative absence of actin stress fibers. 5-AZ increased both the fraction of cells that were positive for invadopodia. We also performed the FITC-gelatin degradation assay in these cells. Degradation of FITC-gelatin was obviously increased after treatment with 5-AZ in ovarian cancer cells (Figure 4B).

### *In vitro* 5-AZ promoted cell migration and invasion in SKOV3 cells

**Figure 5:**
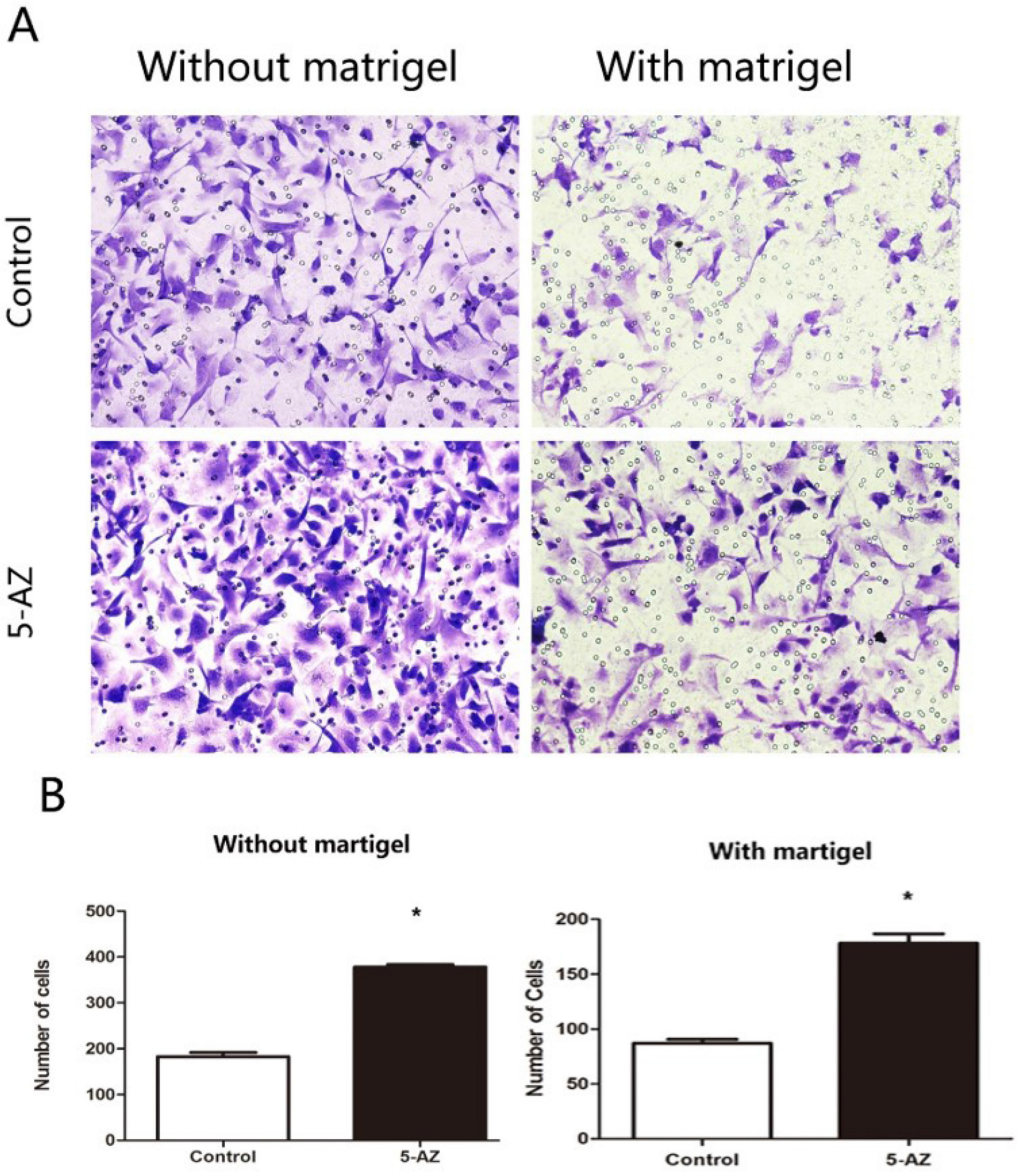
*In vitro* 5-AZ promoted cell migration and invasion by Transwell assays in SKOV3 cells (**A**) SKOV3 cells were untreated (Control, upper) or treated with 5-AZ (30 μM, lower) for 48 h and seeded into Matrigel-uncoated (left panel, transwell Imigration assay), or coated (right panel, transwell invasion assay) Boyden chambers for 8 h. Cells were co-cultured with 10% FBS in the lower and without FBS in the upper of the Boyden chambers. images are from the lower part of the Boyden chambers and are representative of three images taken per condition. (**B**) Quantifications of cells are mean SEM of five independent experiments. The numbers of SKOV3 cells in 5-AZ group seeded with or without Matrigel were both higher than control *P < 0.05.

Invadopodia is always implied with increased invasive and migration potential. To confirm this, migration and invasion of SKOV3 treated with 5-AZ were evaluated, *in vitro*. Untreated (Control) or SKOV3 cells treated with 5-AZ (30 uM) for 48 h and seeded into Matrigel-uncoated (transwell migration assay), or coated (transwell invasion assay) Boyden chambers for 8 h. Cells were co-cultured with 10% FBS in the lower and without FBS in the upper of the Boyden chambers. images are from the lower part of the Boyden chambers and are representative of three images taken per condition. Quantifications of cells are mean SEM of five independent experiments. As shown in Figure 5, the numbers of migrated cells in 5-AZ treated group with or without Matrigel were both more than that in control group, indicating that 5-AZ promoted migration and invasion of the SKOV3 cells, *in vitro*.

### 5-AZ upregulates a pathway related to invadopodia formation, especially the PI3K-AKT pathway

**Figure 6:**
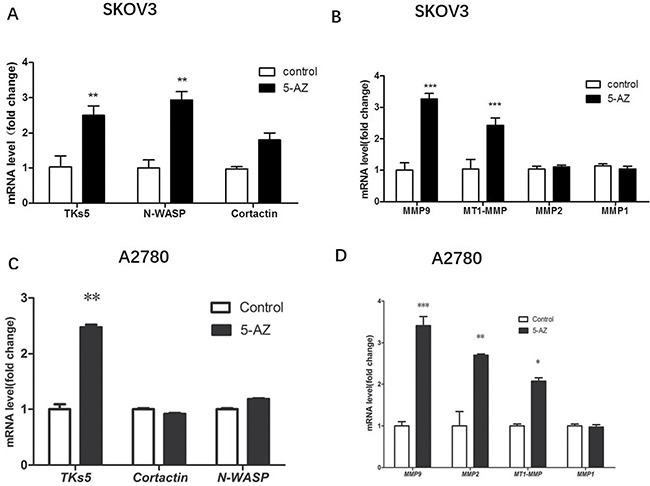
qRT-PCR validation of some invadopodia-related genes are upregulated in the 5-AZ treatment group in SKOV3 and A2780 cells (**A**, **B**) SKOV3 cells that were treated with 5-AZ express high levels of actin-regulating genes (Tks5, N-WASP) that are related to invadopodia formation. The expression of degradation-related genes (MMP9, MT1-MMP) that regulate the function of invadopodia were higher in the 5-AZ treatment group compared with the control group in SKOV3 cells. (**C**, **D**) In A2780 cells, TKs5 and degradation-related genes (MMP9, MMP2, MT1-MMP) were largely upregulated after 5-AZ treatment. Student's *t*-test: mean ± S.E. *n* = 3. *P < 0.05 **P < 0.01 ***P < 0.001. control (untreated cells).

5-AZ increased the number of invadopodia and enhanced the invasion and migration of SKOV3 cells, but the exact pathway that mediates these processes is unclear. To determine the pathways that are linked to 5-AZ treatment and invadopodia formation, qRT-PCR screening of invadopodia-associated genes and selected candidate genes [12, 19–23] was performed to determine the regulated genes. The results confirmed that SKOV3 cells that were treated with 5-AZ express high levels of actin-regulating proteins (e.g., Tks5, N-WASP, Cortactin) [6, 22, 24] (Figure 6A) in addition to degradation-related genes *(e.g*., MMP9, MT1-MMP [6, 7]) compared with control (untreated) cells (Figure 6B). To exclude the possibility that the upregulation is cell line specific, we also sreened the expression of these genes in A2780 cells, since 5-AZ treatment obviously increased the formation of invadopodia in this breast cancer cell lines. As shown in Figure 6C, 6D, in 5-AZ treated A2780 cells, TKs5 and degradation-related genes (MMP9, MMP2, MT1-MMP) were upregulated after 5-AZ treatment, which similar with that in 5-AZ treated SKOV3 cells.

**Figure 7:**
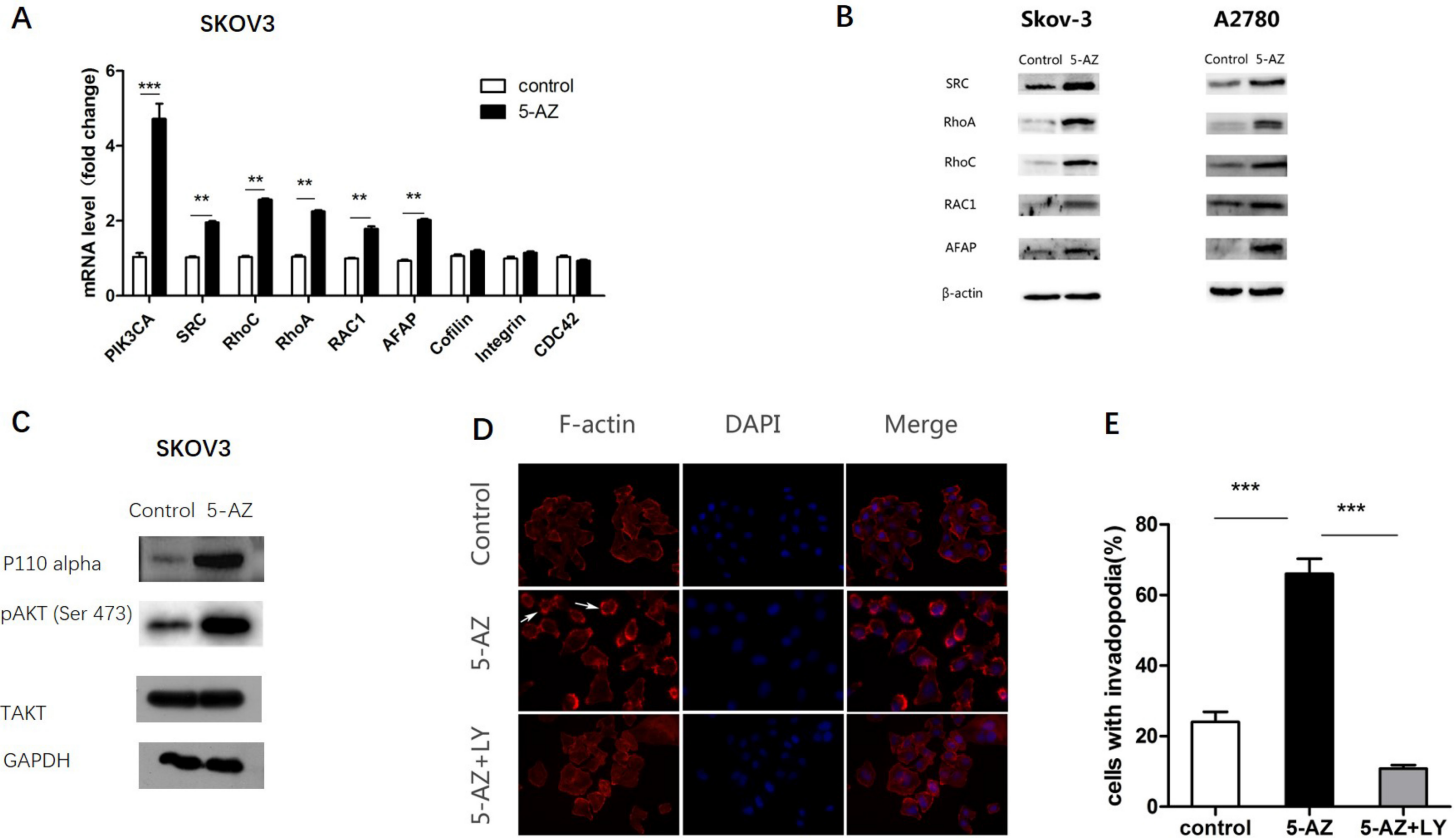
5-AZ upregulates a signaling pathway that is associated with invadopodia, especially the PI3K-AKT pathway (**A**) The upregulation of the mRNA of genes that participate in actin-regulating signaling pathways (e.g., PI3K, SRC, RhoC, RhoA, RAC1, AFAP) and the upregulation of PIK3CA were the most obvious in SKOV3 cells. (**B**) Western blot results confirmed upregulation of actin-regulating signaling pathways (SRC, RhoC, RhoA, RAC1, AFAP) in SKOV3 and A2780 cells. (**C**) Western blot results show an increase of PI3K-pAKT (p110 alpha and pAKT) in the 5-AZ treatment group, but no difference in the T-AKT level compared with the control in SKOV3 cells. (**D**) Images of SKOV3 cells that were treated with DMSO (upper panel), 5-AZ (middle panel) or 5-AZ+ LY294002(PI3K inhibitor 10 μM) (lower panel) for 24 h. Arrowheads point to examples of invadopodia. (**E**) Quantification of cells with invadopodia (left panel) shows inhibition of PI3K leads to an obvious decrease in the number of invadopodia. Student's *t*-test: mean ± S.E. *n* = 3. **P < 0.01 ***P < 0.001.

Moreover, in SKOV3 cells we observed upregulation of genes involved in actin-regulating signaling pathways (e.g., PIK3CA, SRC, RhoC, RhoA, RAC1, AFAP) [25–27] (Figure 7A) and the upregulation of PIK3CA was mostly significant. At the protein level, we confirmed 5-AZ increased the regulating proteins above via Western blot in SKOV3 and A2780 cells (Figure 7B). Consistent with the qRT-PCR data, a Western blot analysis showed that the treatment of cells with 5-AZ resulted in increased levels of phosphorylated AKT and p110 alpha (Figure 7C). Therefore, we concluded that PI3K-AKT pathway was upregulated in cells treated with 5-AZ.

### Inhibition of the PI3K pathway decreases the formation of invadopodia mediated by 5-AZ

**Figure 8:**
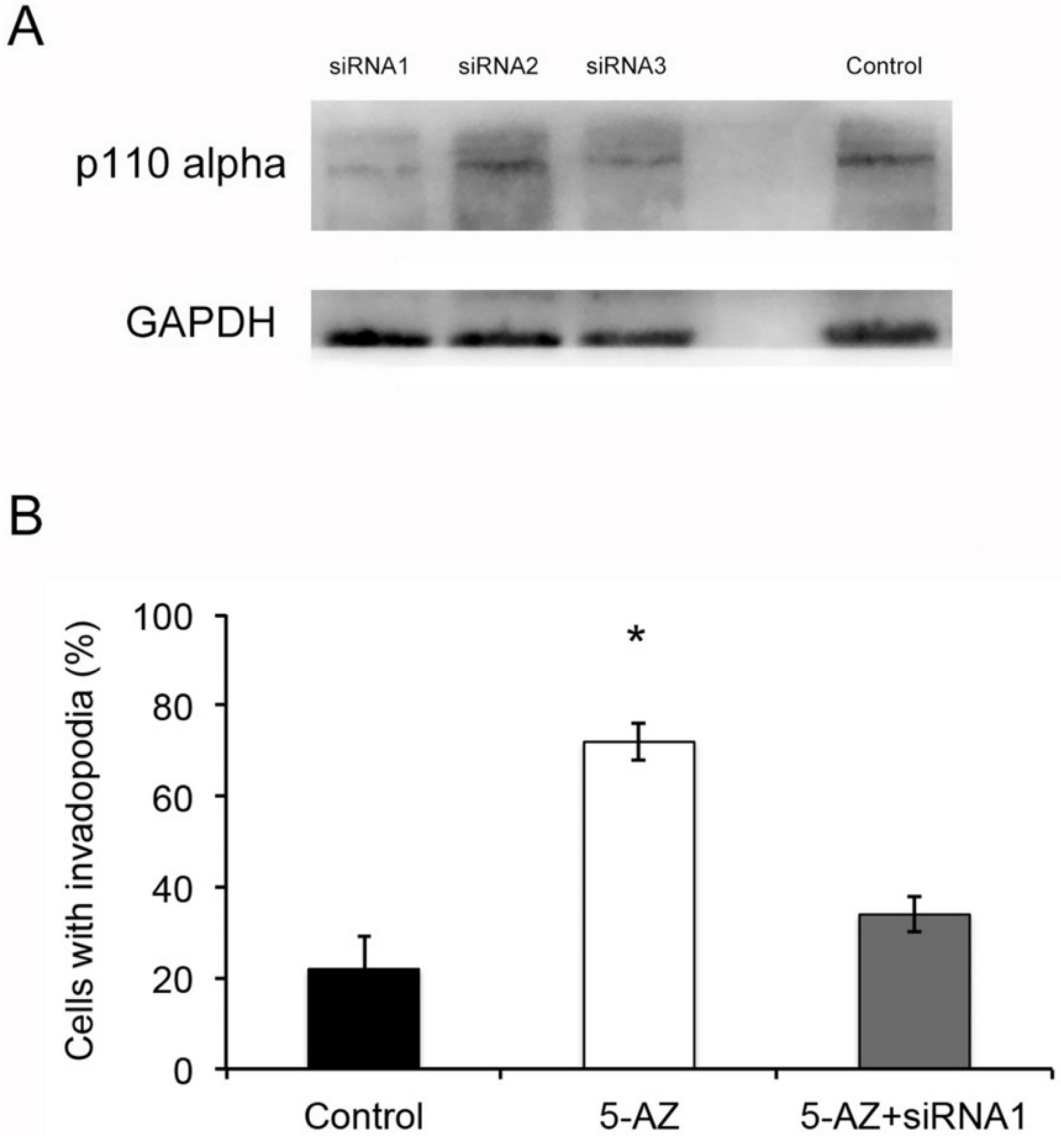
5-AZ increased invadopodia probably via the PI3K-AKT pathway (**A**) PI3KCA siRNA1 successfully inhibited p110 alpha expression compared with other siRNAs. (**B**) Quantification of cells with invadopodia shows there was an obvious decrease in the number of invadopodia in group of 5-AZ and PI3KCA siRNA1 compared with 5-AZ. Student's *t*-test: mean ± S.E. *n* = 3. *P < 0.05.

To test whether 5-AZ could affect the formation and activity of invadopodia though PI3K signaling in SKOV3 cells, we next inhibited PI3K via small molecular LY294002 (10 μM) (a specific PI3K inhibitor) in 5-AZ-treated SKOV3 cells. After 24 h culture of the 5-AZ treated SKOV3 cells, a significant reduction in the number of invadopodia (Figure 7D, 7E) was confirmed. In addition, knock-down of PIK3CA inhibited the invadopodia-promoting capacity of 5-AZ (Figure 8). As shown in Figure 8A, p110alpha subunit of PIK3CA were efficiently knocked-down by a siRNA panel, especially by siRNA1. As a result of PI3K knock-down, the increased invadopodia formation (Figure 8B) by 5-AZ treatment was inhibited, indicating that increased PIK3CA expression accounts for the increased invadopodia formation.

### 5-AZ upregulates PI3K by demethylation of the *PI3K* gene promoter

**Figure 9:**
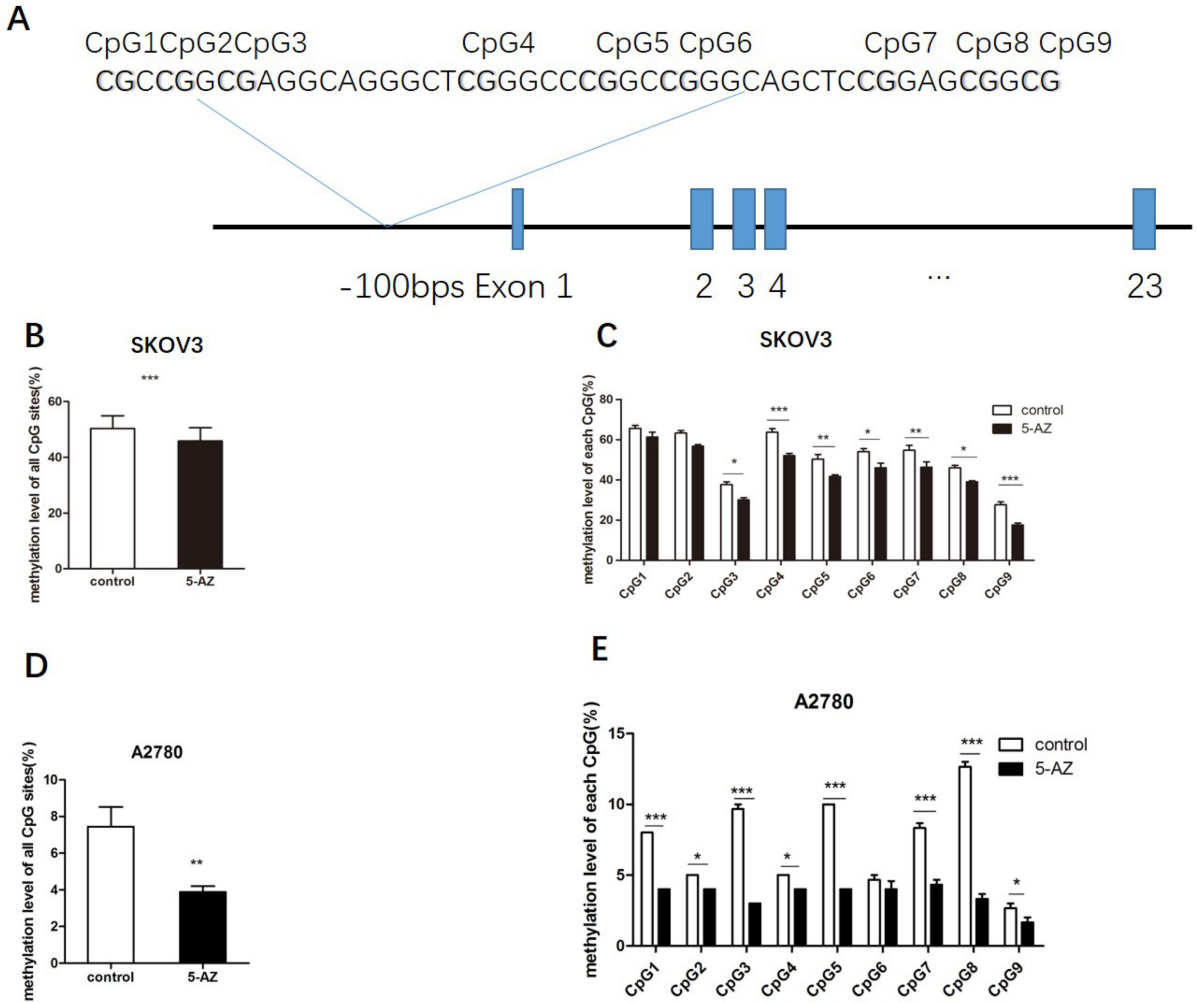
5-AZ upregulates PIK3CA possibly though inhibiting the demethylation of the PIK3CA promoter (**A**) The CpG sites and the single nucleotide polymorphisms in the PI3K gene. The total length of the PIK3CA gene is 91190 bases. RefSeq DNA sequence found in the NCBI GenBank: NC_000003.12. One GC-rich CpG island (nine total CpG sites) (CpG island: −200 bp to 50 bp). (**B**) In SKOV3 cells, the combination of all nine CpG sites and the total mean values of the DNA methylation levels in the PIK3CA gene promoter significantly decreased in the 5-AZ treatment group compared with the control group (49.7% vs. 45.7%). Paired *t*-test (**C**) The DNA methylation levels of each CpG site in the PIK3CA gene promoter were generally lower in the 5-AZ treatment group compared with the control group. The DNA methylation levels at seven CpG sites within the gene (except CpG1 and CpG2) in 5-AZ-treated cells were found to be significantly lower than those in DMSO-treated SKOV3 cells, respectively. (**D**) The total DNA methylation levels had significant difference between the two groups in A2780 (7.4% vs. 3.8%). Paired *t*-test (**E**) The DNA methylation levels at each CpG sites decreased significantly except CpG6 in 5-AZ-treated A2780 cells. Student's *t*-test: mean ± S.E. *n* = 3. *P < 0.05. **P < 0.01. ***P < 0.001.

The PI3K gene is located on chomosome 3q26.3 and comprises 23 exons. MethPrimer software (The Li Lab, San Francisco, CA, USA) was used to predict the promoter regions of the human PIK3CA gene, and we selected one CG-rich CpG island (CpG island: −200 bp to 50 bp). Nine neighboring CpG sites (−100 bp) are located in the promoter region of the PIK3CA gene (Figure 9A). We analyzed PI3K DNA methylation levels not only at each CpG site, but we also analyzed the total methylation level of all the CpG sites The combination of all nine CpG sites and the total mean values of the DNA methylation levels in the PIK3CA gene promoter were increased in the 5-Azacytidine group compared with the control group in SKOV3 (49.7% vs. 45.7%, *P <* 0.001) and A2780 cells (7.4% vs 3.8%, *P <* 0.001) (Figure 9B, 9D). DNA methylation patterns of each CpG site in the PIK3CA gene promoter region of SKOV3 and A2780 cells with and without 5-Azacytidine treatment were presented (Figure 9C, 9E). Both in SKOV3 and A2780 cells, the DNA methylation levels of all the CpG sites in the PIK3CA gene promoter were both lower in the 5-AZ treated group compared with the control (Supplementary Figure 5). The DNA methylation levels at seven CpG sites within the gene (except CpG1 and CpG2) in 5-Azacytidine-treated SKOV3 cells (CpG3 37.6% vs. 30%, *P <* 0.05; CpG4 61.6% vs. 52%, *P <* 0.001; CpG5 50.3% vs. 41.6%, *P <* 0.01; CpG6 54%vs. 46%, *P <* 0.05; and CpG7 54.6% vs. 46.3%, *P <* 0.01 CpG8 46% vs. 39%, *P <* 0.05, CpG9 26.7% vs. 18.7%, *P <* 0.01) (Figure 9C) and eight CpG sites (except CpG6) in 5-AZ treated A2780 cells(CpG1 8.6% vs. 4.2%, *P <* 0.001; CpG2 5.6% vs. 4%, *P <* 0.05; CpG3 9.6% vs. 3.1%, *P <* 0.001; CpG4 5.4%vs. 4.1%, *P <* 0.05; CpG5 10.6% vs. 4.3%, *P <* 0.001; CpG7 8.3% vs. 4.3%, *P <* 0.001; CpG8 12.7% vs. 3.3%, *P <* 0.001; CpG9 2.6% vs. 1.6%, *P <* 0.05) were found to be both lower than those in the control groups, respectively. These data indicated that 5-AZ potentially regulated the expression of *PIK3CA* via demethylating of its promoter.

## DISCUSSION

In this study, we found that 5-AZ increased the formation of invadopodia, enhanced their function and promoted distant metastasis of ovarian cancer. Interestingly, we determined that the PI3K pathway quantitatively and functionally promoted the formation of invadopodia. Further, the demethylation of the PI3K promoter by 5-AZ contributed to the upregulation of PI3K-related pathways.

DNA demethylating agents are nucleoside analogues that are designed to reduce DNA methylation of TSGs, but they exert a wide influence on multiple genes and do not necessarily have a specific target. Given that the demethylation effect of 5-AZ is nonspecific, there are concerns regarding the potential stimulation of protumoral genes, such as a subset of the MMP genes [28]. It is therefore important to understand how cells tightly regulate invadopodia formation, especially when this occurs by epigenetic mechanisms, to modulate their invasive properties. In this study, we focused on the role of a DNA demethylating agent in the regulation of the invasive and metastatic behavior associated with the modulation of invadopodia in human SKOV3 cells. To evaluate the relevance of this observation, we have examined the effect of 5-AZ on invasive and metastatic nodules of human EOC cell xenografts. We demonstrated that 5-AZ increased the number of invasive and metastatic nodules on the peritoneal surfaces of SKOV3 xenografts *in vivo* although a little slight decrease was observed in the average volume of each nodule and in the tumor weight between the two groups.

The lethal outcome of the vast majority of cancers is due to the dissemination of metastatic tumor cells and the outgrowth of secondary tumors at distant sites. Invadopodia are implicated in the invasion and metastasis processes because they initiate the degradation of ECM components and because they regulate cancer cell migration [29, 30]. Invadopodia and podosomes are protrusive structures that are associated with ECM degradation and invasive cellular migration [7]. To verify the invasive behavior that was enhanced by 5-AZ treatment of cancer cells, we explored the effect of 5-AZ on invadopodia in EOC cells. We stained for F-actin to demonstrate that 5-AZ promoted invadopodia formation and used a gelatin degradation assay to demonstrate that 5-AZ promoted the function of invadopodia. The change in invadopodia was reflected not only in the number of invadopodia-positive cells but also in the number of invadopodia per cell. We show here that 5-AZ treatment promotes invasive and metastatic behavior of SKOV3 cells via the stimulation of invadopodia formation. These results raise concern that continued treatment of patients with epithelial ovarian cancer, and specifically those with refractory tumors, with 5-AZ may stimulate tumor cell extravasation and dissemination. Furthermore, most patients with EOC initially respond to surgery, chemotherapy and 5-AZ treatment but then relapse and become refractory to other therapies; moreover, these patients typically have more aggressive tumors that are associated with metastasis into the abdomen [31–33].

Previous reports have revealed that exposure to 5-AZ increased the invasive properties of several types of cancer cells including pancreatic [34] and acute myeloid leukemia cells [28] *in vitro* via the reactivation of MMP genes. The mRNA expression of invadopodia-associated genes (e.g., Tks5, Cortactin, N-WASP) and MMP genes (MMP9, MMP14, but not MMP1, MMP2) was significantly increased in the SKOV3 cells that were treated with 5-AZ compared with control cells. This demonstrated that 5-AZ promoted the formation and function of invadopodia not only reactivated MMP genes. Importantly, qPCR results demonstrated strong upregulation of gene members of several actin-regulating signaling pathways (PI3K, SRC, RhoC, RhoA, RAC1, AFAP). We then focused on the upregulation of PI3K because the change in PI3K expression was the most obvious and because previous reports identified that PI3K signaling via p110 alpha regulates invadopodia-mediated invasion of breast cancer and ovarian cancer cells [35]. Western blot analyses showed that p110 alpha and p-AKT proteins were expressed at higher levels in 5-AZ-treated cells, whereas the expression of total AKT protein was not different between the two groups. In addition, we found that in the ovarian cancer cells that were treated with 5-AZ, invadopodia formation was clearly blocked by treatment with a PI3K inhibitor (LY294002). This suggests that 5-AZ activates the PI3K pathway though the upregulation of PI3K but not AKT.

Universal hypomethylation of the entire genome and hypomethylation of specific sites in the promoter regions of oncogenes increase cancer risk. The lower degree of methylation in proto-oncogenes is closely related to invasiveness [36]. Azacytidine is a hypomethylating agent that has been shown to induce the re-expression of many tumor suppressor genes and some oncogenes in cancer cells. To date, few investigations have found a relationship between the effect of 5-AZ and PIK3CA methylation in ovarian cancer, especially with the invadopodia-promoting function of PI3K. As PI3K signaling regulates invadopodia-mediated invasion of ovarian cancer cells, which is consistent with data from previous studies [37], we consequently analyzed the methylation levels of the promoter of the PI3K gene. This allowed us to explore whether 5-AZ upregulated PI3K though the inhibition of DNA methyltransferases. We found that the DNA methylation levels of all CpG sites in the promoter of the PI3K gene were both lower in the 5-AZ-treated ovarian cancer cells compared with the control cells although the difference was small. Moreover, this difference was statistically significant in seven of the nine CpG sites. Thus, we believe that PIK3CA hypomethylation may play a role in the activation mechanism of the PI3K/AKT pathway and that hypomethylation is closely related to the effects of 5-AZ. We only selected oneCpG island based on the CpGplot program. The full effects of PIK3CA methylation may not be completely apparent due to missing methylation sites. The detection of any additional potential sites is part of our ongoing research. In the future, we will obtain additional samples and examine other CpG islands.

## MATERIALS AND METHODS

### Animal xenograft studies

All protocols for the animal experiments were approved by the Sichuan University Animal Care and Use Committee and were performed in accordance with Sichuan University Guidelines for Experimental Animals (2012 Edition) and Sichuan University Guidelines for Pre-Clinical Research for Drugs. Female nude mice were purchased from the Sichuan University Research Animal Resources (Chengdu, Sichuan Province, China). All xenografts were established from cells (5 × 10^6^ SKOV3 cells in a total volume of 100 μL) that were delivered by intraperitoneal (IP) injection into the abdomen of 6–7-week-old female mice. The tumor volume was calculated as (L × W2)/2. Then, after two days of tumor cell injection, *5-A*Z was administered via IP injection at 2 mg/kg to mice in the treatment group, while the same volume of normal saline (NS) was administered to mice in the control group thrice weekly for 10 weeks. The vehicle for 5-AZ was Dulbecco's Modified Eagle's Medium (DMEM). All other vehicle injections were composed of saline. All animals were sacrificed when mice in the NS group began to die. The weight of each excised peritoneal metastasis was evaluated, and the number of metastatic nodules in the peritoneum was counted.

### Cell culture

The human cancer cell line was purchased from the American Type Culture Collection (ATCC; Manassas, VA, USA). SKOV3, A2780, MDA-MB 231 and MCF-7 cells were grown in DMEM, supplemented with 10% fetal bovine serum (FBS, Gibco, Grand Island, NY, USA) and penicillin/streptomycin (Gibco); all cells were grown at 37°C in a 5% CO2 atmosphere.

### Assays to determine the formation and function of invadopodia

SKOV3 cells were nutrient-starved on glass coverslips and processed for immunofluorescence 48 hours after *5-*AZ treatment. The quantification of invadopodia was performed on at least 15 randomly selected fields that represented ∼150 cells per experimental point. SKOV3 cells with at least one complete rosette of invadopodia were scored as positive. Total cell numbers were calculated by scoring the number of nuclei in the same field. Invadopodium function assays were performed as previously described [24]. Fluorescein isothiocyanate (FITC)-labeled gelatin-coated coverslips were prepared with some modifications, and SKOV3 cells were cultured such that by 24 h the coverslips contained nearly confluent cells. The coverslips were subsequently processed using standard fluorescence microscopy procedures. The quantification of gelatin degradation activity was performed on at least 15 randomly selected fields that represented a minimum of 200 total cells per experimental point. The quantification of the degraded areas per field was performed with Image J software version 1.34 (http://rsb.info.nih.gov/ij/), and the percentage of degraded areas per field was normalized to the number of cells in that field.

### Fluorescence staining

Immunofluorescence was performed on paraformaldehyde-fixed cells. First, the cells were permeabilized with 0.1% Triton X-100 in phosphate-buffered saline (PBS) and then blocked with 0.1% Triton X-100 and 5% bovine serum albumin (BSA) in PBS for 1 h followed by a wash. Actin was visualized with Alexa Fluor 594-conjugated phalloidin, and the nuclei were stained with DAPI. Coverslips were coated with poly-l-lysine and fixed with glutaraldehyde. The slides were then covered with 500 μl of Alexa Fluor 488-coupled gelatin (0.02% porcine gelatin Alexa Fluor 488, 0.1% porcine gelatin, 0.2% sucrose; Invitrogen) for 3 h at 37°C in the dark. The slides were sterilized with ethanol, rinsed with PBS, and medium was added. The cells were then added and allowed to adhere for 24 h. After the cells were labeled with rhodamine-phalloidin, immunofluorescence observed using a confocal microscope (LSM510META; Carl Zeiss, Jena, Germany). For real-time imaging, the cells were seeded onto a glass-bottom Petri dish (MatTek, Ashland, MA) in Leibovitz's 15 media (Invitrogen). Images were obtained after 1 h so that the cells time had time to flatten in the dish. The ambient temperature was maintained at 37°C with a thermostat sealed chamber around the confocal microscope. Images were obtained with a confocal microscope (LSM510META).

### Knock-down of p110alpha by siRNA

Knock-down of p110alpha was accomplished with RNA interference with siRNA. The siRNA kit containing three different siRNA targeting p110alpha were purchased from RiBoBio (Guangzhou, China). RNAi interferences were exerted according to the manufacturer's manual. Efficiency of RNAi were determined the p110alpha expression level detected by Western Blotting.

### Quantitative reverse transcription real-time polymerase chain reaction (qRT-PCR)

qRT-PCR was performed using human primers Table 1 and SYBR Green Master Mix (Applied Biosystems, Foster City, CA). Briefly, total RNA was isolated from the cells with an RNeasy Mini Kit (QIAGEN, Valencia, CA), and complementary DNA was subsequently generated using the Super-Script III First-strand Synthesis System (Invitrogen, Carlsbad, CA). The polymerase chain reaction (PCR) conditions were as follows: 10 min at 95°C for AmpliTaq Gold activation, followed by 40 cycles at 95°C for 15 s for denaturation and then 60°C for 1 min for annealing/extension. The RT–PCR was performed in a MyiQ single-color real-time PCR detection system (Bio-Rad, Hercules, CA) (Supplementary Figure 4). Relative quantification was performed with glyceraldehyde-3-phosphate dehydrogenase (GAPDH) transcript as an endogenous housekeeping control. PCR products were separated on a 1% agarose gel and were imaged under UV light. The expression of GAPDH was chosen as an internal control. Expression level of interested genes in control was set as 1 and fold change in the experimental groups compared with control was calculated. Sequence of the Primers were obtained from primer bank and blasted all primers in gene bank.

**Table 1:** List of primers for qRT-PCR assay

Gene symbol	Sequence (5′→3′)
GAPDH	F: GGAGCGAGATCCCTCCAAAAT R: GGCTGTTGTCATACTTCTCATGG
β-actin	F: CATGTACGTTGCTATCCAGGC R: CTCCTTAATGTCACGCACGAT
Tks5	F: AGACTATCTACCGGAGGTACAGC R: GCCACCTTCAATGGGAAACTT
N-WASP	F: GATGCTTGGACGAAAATGCTTG R: CCCCACAATGCTCCTTGGT
Cortactin	F:GCTTTGAGTATCAAGGCAAAACG R: CCAAGGGCACATTTGTCTTGT
MMP9	F: TGTACCGCTATGGTTACACTCG R: GGCAGGGACAGTTGCTTCT
MMP14	F: GGCTACAGCAATATGGCTACC R: GATGGCCGCTGAGAGTGAC
MMP2	F: TACAGGATCATTGGCTACACACC R: GGTCACATCGCTCCAGACT
MMP1	F: CTCTGGAGTAATGTCACACCTCT R: TGTTGGTCCACCTTTCATCTTC
PIK3CA	F: CCACGACCATCATCAGGTGAA R: CCTCACGGAGGCATTCTAAAGT
SRC	F: GACAGGCTACATCCCCAGC R: CGTCTGGTGATCTTGCCAAAA
RhoA	F: GGAAAGCAGGTAGAGTTGGCT R: GGCTGTCGATGGAAAAACACAT
RhoC	F: GGAGGTCTACGTCCCTACTGT R: CGCAGTCGATCATAGTCTTCC
RAC1	F: ATGTCCGTGCAAAGTGGTATC R: CTCGGATCGCTTCGTCAAACA
AFAP	F: AGGAGACCGCTAACAGCCT R: GCTCATCGCATCGGAATCATAA
Coffilin	F: TACGCCACCTTTGTCAAGATG R: CCTTGGAGCTGGCATAAATCAT
Integrin	F: CCTACTTCTGCACGATGTGATG R: CCTTTGCTACGGTTGGTTACATT
CDC42	F: CCATCGGAATATGTACCGACTG R: CTCAGCGGTCGTAATCTGTCA

### Western blot

The cells were harvested, and all soluble proteins were run on polyacrylamide gels and transferred onto nitrocellulose membranes. The membranes were blotted for relevant proteins using specific primary antibodies (Supplementary Figure 3, as described for each experiment). Secondary antibodies were FITC- or horseradish peroxidase-conjugated; fluorescence was detected with a Typhoon 9410 scanner (GE Healthcare Life Sciences, Piscataway, NJ) or with enhanced chemiluminescence detection reagent. Quantification of the digital images obtained was performed using ImageQuant 5.2 software (GE Healthcare Life Sciences).

### Transwell assay

SKOV3 cells were untreated or treated with 5-AZ (30uM) for 48 h and seeded into Matrigel-uncoated (transwell Imigration assay), or coated (transwell invasion assay) Boyden chambers for 8 h. Cells were co-cultured with 10% FBS in the lower and without FBS in the upper of the Boyden chambers. images are from the lower part of the Boyden chambers and are representative of three images taken per condition.

### Bisulfite treatment and pyrosequencing

An epigenetic analysis was performed by bisulfite pyrosequencing, which is a sensitive and accurate method for the detection of methylation. DNA was treated with sodium bisulfite using the EpiTect Bisulfite Kit (Qiagen), which was followed by the cleanup of bisulfite-converted DNA. PCR amplification was then performed with a PyroMark CpG Assay Kit (Qiagen) and a PyroMark Gold Q96 Reagent Kit (Qiagen) in a PyroMark Q96 system (Biotage AB, Uppsala Sweden). PyroMark PCR master mix includes HotStarTaq DNA polymerase and optimized PyroMark reaction buffer that contains 3 mM MgCl2 and dNTPs, 10x CoralLoad Concentrate, 5x Q-Solution, 25 mM MgCl2, and RNase-free water. The PCR amplicon covers the sequence of human chomosome 3q: NC_000003.12 (179148114.179240084). Nine CpG sites are located in the promoter region of the *PIK3CA* gene (-100 bps) as indicated with the bold letter ‘**C**’ and recorded as CpG1-9 (Figure 6A). Finally, the methylation levels of these CpG sites were detected with a PyroMark Gold 96 Reagent Kit (Qiagen) and a PyroMark Q96 ID pyrosequencing system (Biotage). The unmethylated and unconverted DNA samples (Qiagen) were used to control the conversion efficiency during the bisulfite treatment and the accuracy of the methylation analyses. PyroQ-CpG software (Biotage) was used for methylation data analysis.

### Statistical analysis

Unless otherwise noted, for all experiments that contained single comparisons, 1-way ANOVA with Newman–Keuls post-hoc tests were used. For grouped samples, 2-way ANOVA with Bonferroni post-hoc tests were performed with GraphPad Prism(San Diego, CA). All graphs are the mean ± SEM (*P < 0.05; **P < 0.01; ***P < 0.001).

## CONCLUSIONS

In conclusion, the present study provides evidence that 5-Azacytidine, a hypomethylating agent, could promote tumor metastasis and invadopodia formation through the upregulation of PI3K in ovarian cancer cells. The use of inhibitors of methylation could, at least in some cases, stimulate the invasive growth or metastasis of ovarian cancer though reactivation of PIK3CA genes that are silenced by promoter methylation. This potential effect of epigenetic factors on tumor progression provides rationale for therapies that inhibit PI3K-invadopodia-mediated invasion and metastasis.

## SUPPLEMENTARY FIGURES


