# Supplementary material for: 5-Azacytidine promotes invadopodia formation and tumor metastasis through the upregulation of PI3K in ovarian cancer cells
Source: Oncotarget. 2017 Jun 20;8(36):60173–87. doi: 10.18632/oncotarget.18580 (PMC5601130; doi:10.18632/oncotarget.18580)
Supplement: Supplementary file 1 [file oncotarget-08-60173-s001.pdf]

5-Azacytidine promotes invadopodia formation and tumor metastasis through the upregulation of PI3K in ovarian cancer cells

Supplementary Materials

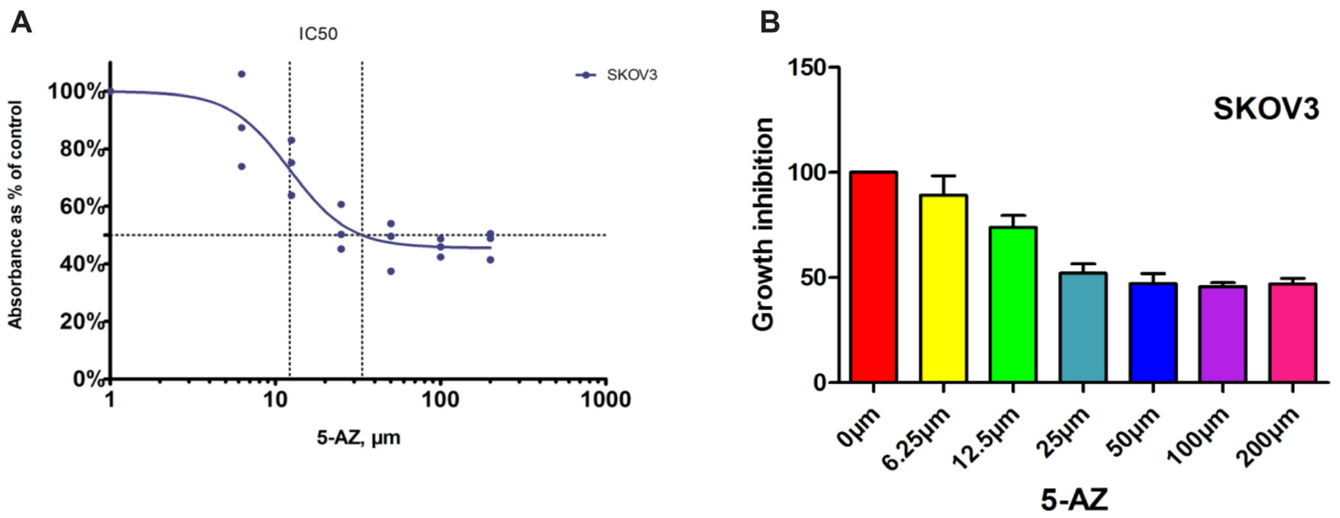

Supplementary Figure 1: Growth Inhibition of DNA Methylation Inhibitor for SKOV3 Ovarian Cancer cells *in vitro*. (**A**) IC50 indicates 50% inhibitory concentration. IC50 of 5-AZ was30μm/l. (**B**) 5-AZ inhibited cell growth at different concentrations. Student's *t*-test: mean ± S.E. *n* = 3.

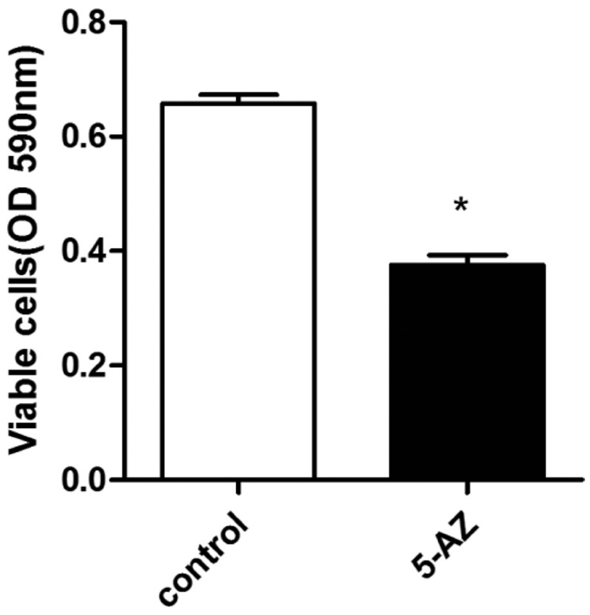

Supplementary Figure 2: SKOV3 cell growth was inhibited by 5-AZ(30 μm/l) treatment. Student's *t*-test: mean ± S.E. *n* = 3. *P* = 0.012.

The information of antibodys used in this article.

Antigen	Code	Host	Dilution	Manufacture
β-actin	sc-69879	mouse	1:5000	Santa Cruz Biotechnology,CA
GAPDH	sc-47724	mouse	1:3000	Santa Cruz Biotechnology,CA
PIK3α	ab40776	Rabbit	1:1000	abcam, Cambridge, UK
AKT	#9272	Rabbit	1:1000	Cell Signaling,Beverly,MA
p-AKT	#4060	Rabbit	1:2000	Cell Signaling,Beverly,MA
SRC	ab109381	Rabbit	1:1000	abcam, Cambridge, UK
Rac1	ab155938	Rabbit	1:1000	abcam, Cambridge, UK
AFAP	ab72035	mouse	1:500	abcam, Cambridge, UK
RHOC	ab54837	mouse	1:500	abcam, Cambridge, UK
RhoA	ab54835	mouse	1:500	abcam, Cambridge, UK
Rabbit IgG	ZB-2301	Goat	1:10000	zsbio,Beijing,CN
Mouse IgG	ZB-5305	Goat	1:10000	zsbio,Beijing,CN

Supplementary Figure 3: The information of all antibodies used in this article.

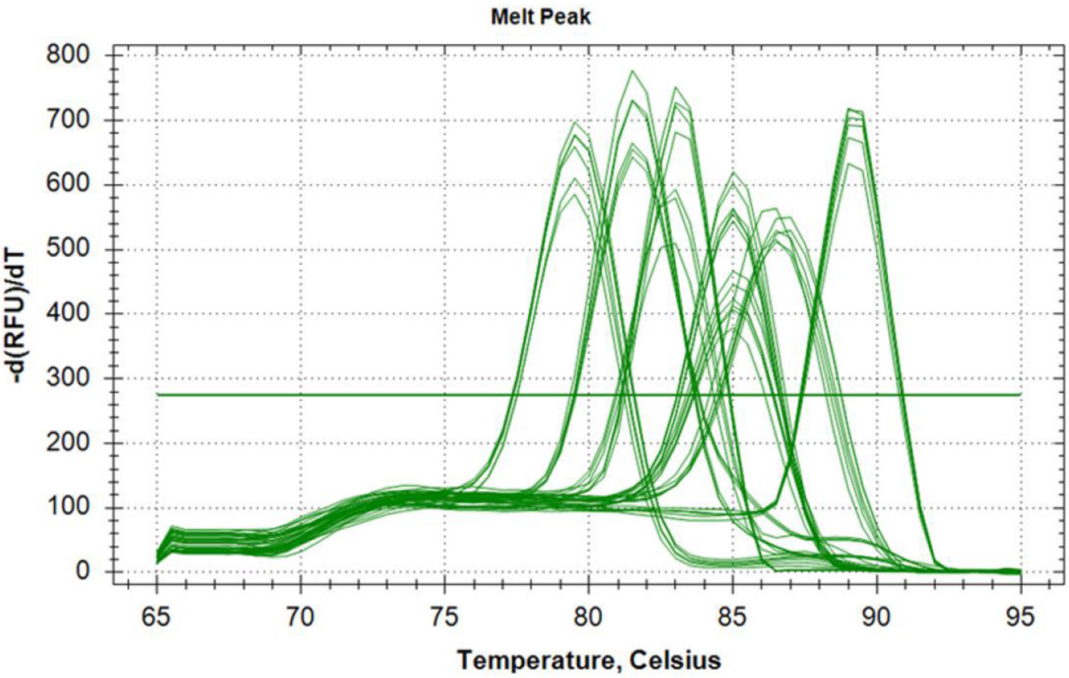

Supplementary Figure 4: The Melt Peak of qRT-PCR in the process of assessed the expression of different genes. All the primers we designed having similar melting temperatures and they can be performed in a MyiQ single-color real-time PCR in stated denaturing, annealing and extending conditions with high specificity in CFX96TM Real-Time System (BIO-RAD, Hercules, CA).

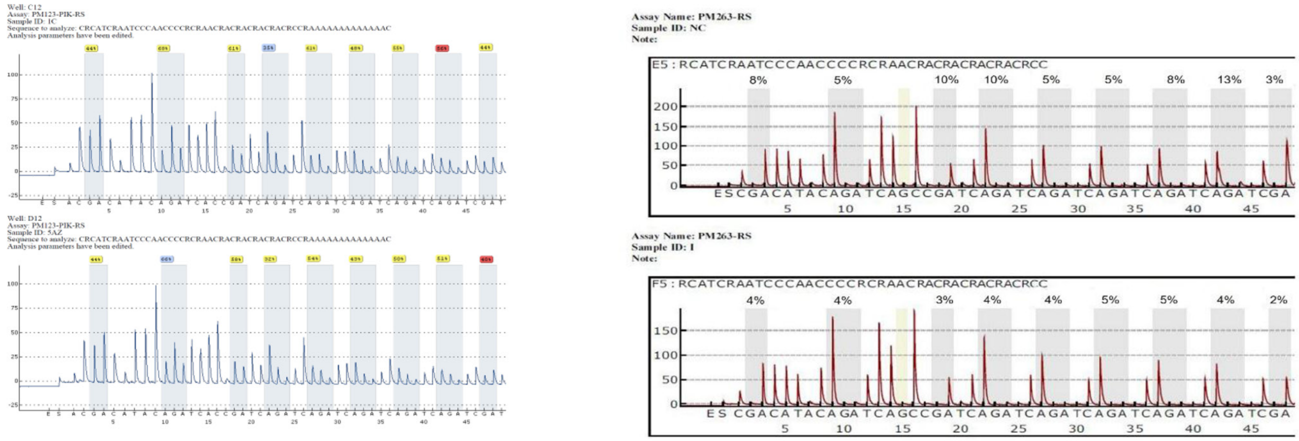

Supplementary Figure 5: Original pyrosequencing data in SKOV3 and A2780. (**A**) Methylation of each CpG island between Sample IC(control) and sample 5-AZ in SKOV3. (**B**) Methylation of each CpG island in sample NC(control) compared with sample 1(5-AZ) in A2780.
